# Effect of comfort care combined with family participatory care during the perioperative period of cesarean section in obstetrics and gynecology

**DOI:** 10.1093/fampra/cmaf091

**Published:** 2025-12-02

**Authors:** Chunyan Wan, Ruihuan Wang, Qing Liu, Mei Xu

**Affiliations:** Hemodialysis Room, Qingdao Chengyang People's Hospital, Qingdao, Shandong 266109, China; Department of Medical Imaging, Qingdao Chengyang People's Hospital, Qingdao, Shandong 266109, China; Hemodialysis Room, Qingdao Chengyang People's Hospital, Qingdao, Shandong 266109, China; Delivery Room, Qingdao Chengyang People's Hospital, Qingdao, Shandong 266109, China

**Keywords:** comfort care, family participatory care, cesarean section, obstetrics and gynecology, perioperative period, maternal postoperative pain, maternal self-care ability, nursing satisfaction

## Abstract

**Objective:**

To explore the effects of comfort care and family participatory care during the perioperative period of cesarean section in obstetrics and gynecology.

**Methods:**

This single-center, randomized controlled trial assigned 90 cesarean women to two groups: control (conventional perioperative care) and observation (comfort and family participatory care). The primary outcome measures included maternal pain [Visual Analog Scale (VAS)], anxiety [Self-Rating Anxiety Scale (SAS)], and depression [Self-Rating Depression Scale (SDS)]. Secondary outcomes included mean arterial pressure and heart rate, adrenaline and cortisol levels, lactation onset time, 24-h postpartum lactation volume, off-bed activity time, anal defecation time, and hospital stay, as well as evaluating maternal self-care ability [Exercise of Self-Care Agency Scale (ESCA)], maternal complications, and maternal satisfaction with nursing assessed using a satisfaction questionnaire at discharge.

**Results:**

The observation group possessed lower VAS at 24, 48, and 72 h postoperatively, and SAS and SDS scores postoperatively, lower mean arterial pressure, heart rate, and adrenaline and cortisol levels, higher scores of self-care skills, self-care responsibility, self-concept, and self-care health knowledge, shorter times to lactation onset, off-bed activity, anal defecation, and hospital stay, greater 24-h postpartum lactation volume, lower incidence rate of complications (4.44% vs 24.44%), and higher nursing satisfaction (97.78% vs 84.22%) compared with the control group (all *P* < 0.05).

**Conclusion:**

During the perioperative period of cesarean section, comfort care and family participatory care can effectively alleviate postoperative pain, reduce anxiety and depression, improve stress responses, enhance maternal self-care ability and nursing satisfaction, and decrease the incidence of complications.

Key messages

- Comfort care and family participatory care reduce postoperative maternal pain
- Comfort care and family participatory care reduce maternal SAS and SDS scores
- Comfort care and family participatory care reduce maternal mean arterial pressure

## Introduction

Obstetrics and gynecology is a specialty area where nurses are able to exert positive effects on women's health and operative results. Perinatal nurses continue to take the lead in making cesarean section a patient- and family-centered event when there are indications for cesarean section in clinical practice [1]. The rate of cesarean section is progressively increasing in many parts of the world [2]. When medically indicated, cesarean section can be utilized as a life-saving intervention, but this intervention can also result in short-term or long-term health effects for mothers and children [3]. The perioperative period represents a potential data-rich environment to optimize patients’ health through targeted prehabilitation [4].

Comfort can be regarded as a primary patient objective and is central to patient experience, and comfort maximization is a universal goal of healthcare staff. The comfort theory, developed by Kolcaba, is commonly known for its systematization and projection regarding comfort care [5]. The management of comfort is a priority for patients in all settings. The comfort theory is reported to provide foundational and holistic approaches to manage comfort [6]. Within the relevant conceptual framework, comfort care centers around meeting patients’ comfort needs in physiological, psycho-spiritual, environmental, and socio-cultural contexts, aiming to achieve its goals through the implementation of therapeutic interventions that enhance comfort (i.e. comfort care intervention strategies) [5]. The comfort theory is implemented in nursing care for new mothers, which can not only provide an applicable concept that facilitates the clinical nursing care of women during the postpartum period but also help raise their comfort level [7]. It is demonstrated that nursing care based on the comfort theory for women undergoing cesarean section meets their comfort needs and increases their postpartum comfort levels [8]. The application of comfort care in inpatient nursing can enable parturients to enjoy the joy of new motherhood in a comfortable and warm environment, guide them to become familiar with relevant nursing knowledge, increase the success rate of breastfeeding as soon as possible, and promote the healthy growth of newborns [9]. It not only helps alleviate surgical pain and facilitate patient recovery but also reduces the incidence of postoperative complications [10]. The participation of family in nursing care programs is of great significance to the outcomes of patients [11]. Patient- and family-centred care refers to care arranged around patients’ and their relatives’ beliefs and needs, which is expected to positively influence the quality of care [12].

Based on the aforementioned studies, we have come to realize that previous research in the fields of obstetrics and gynecology has mostly focused on examining the effects of comfort care or family involvement independently, with a lack of validation regarding their synergistic effects. This study innovatively integrates the four dimensions of comfort care (physiological, psychological, environmental, and social) with family participatory care to form a holistic model that covers the entire perioperative process. It aims to clarify the effectiveness of applying this nursing model during the perioperative period of cesarean sections in obstetrics and gynecology, fill the gap in existing research concerning the synergistic effects of multidimensional nursing, provide a replicable and scalable standardized protocol for perioperative nursing in cesarean sections, and ultimately improve maternal and infant outcomes while enhancing the quality of nursing services.

## Materials and methods

### Ethics statement

The study was under the approval of the Ethics Committee of Qingdao Chengyang People's Hospital. Mothers and their families gave informed consent to the study and signed the written consent form.

### Study design and subjects

This study was a single-center, randomized controlled trial. Ninety cases of parturient women who underwent cesarean section in Qingdao Chengyang People's Hospital from March 2019 to March 2021 were selected as study subjects.

**Figure 1.**
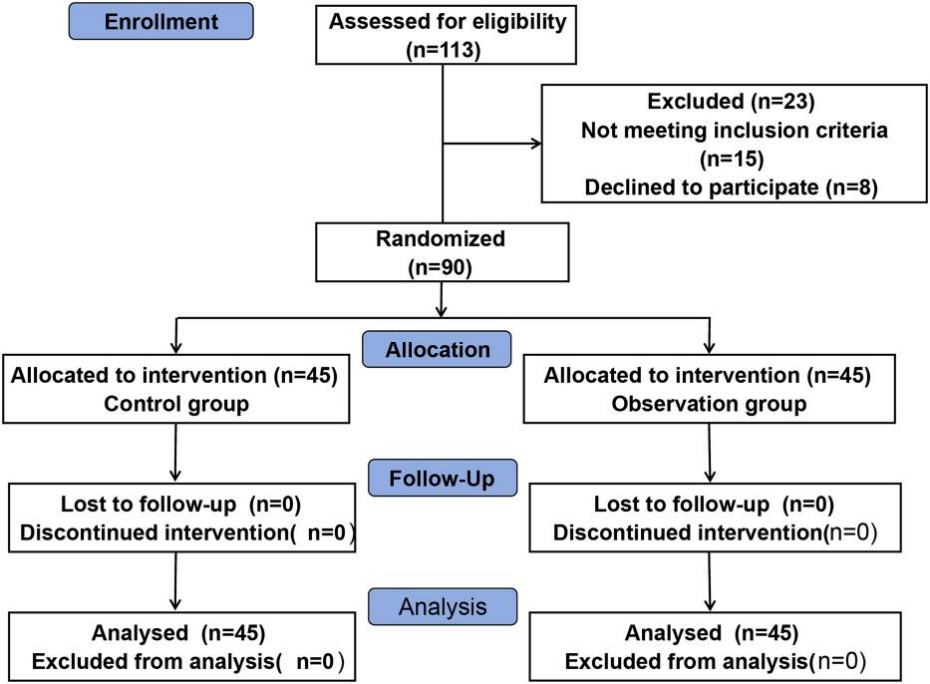
CONSORT flow diagram.

Inclusion criteria: (i) parturient women with a single fetus and in full-term pregnancy; (ii) those who met the indications for cesarean section, who underwent cesarean section after failed vaginal trial of labor or underwent cesarean section directly; (iii) those with complete clinical data; and (iv) parturient women and their families signed the informed consent form. Exclusion criteria: (i) those combined with malignant tumors and hematologic diseases; (ii) those with physical dysfunction; and (iii) those with mental abnormality or poor nursing cooperation. Figure 1 presents the CONSORT flow diagram illustrating the recruitment of study participants.

### Sample size calculation

In this study, the pain score of the research subjects was used as the primary outcome measure. The sample size was estimated using the formula for calculating the mean of two independent samples based on the formula $n\;=\;\frac{2\;{\left(Z_{\alpha }\;+\;Z_{\beta }\right)}^{2}*\sigma^{2}}{\delta^{2}}$. In the formula, *n* represents the sample size per group, *σ* denotes the sample standard deviation, and *δ* indicates the difference in means between the two groups. With a two-sided *α* set at 0.05, *β* at 0.2, and a power (1−*β*) of 0.8, reference tables indicate that *Z_α_* = 1.96 and *Z_β_* = 0.84. By consulting relevant literature [13], where *σ* = 0.76 and *δ* = 0.48, the sample size per group was calculated as *n* = 39. A 1:1 randomization ratio was adopted, with 39 subjects required in both the observation group and the control group. Taking into account a 15% sample attrition rate, 45 subjects were needed for each group, resulting in a total of 90 subjects ultimately included in the study.

### Randomization and blinding

Mothers were grouped using the random number table method. Ninety numbers were selected from the random number table and divided into two groups in sequence, with the first group of 45 numbers labeled as A and the second group of 45 numbers labeled as B. These 90 labeled random numbers were then placed in ascending order into sealed, opaque envelopes numbered from 1 to 90. Participants were allocated to groups based on their order of admission; mothers in envelopes marked A were assigned to the control group, while those in envelopes marked B were assigned to the observation group. Grouping and intervention were strictly conducted according to the markings inside the envelopes. This study did not employ blinding for the research subjects and investigators, but blinding was implemented for personnel involved in data collection, data analysis, and outcome assessment.

### Nursing methods

In the control group, routine perioperative care was administered: Mothers were routinely provided with psychological support and health education before surgery, and were instructed, along with their families, on surgical and postoperative preparations. During surgery, close attention was paid to the mothers’ vital signs. Postoperatively, wound observation and dressing changes were performed, along with routine anti-infective and pain management care, with a focus on preventing postoperative complications. Mothers and their families were informed of precautions, encouraged to engage in early ambulation, and provided with postpartum feeding education.

In the observation group, comfort care combined with family participatory care was implemented during the perioperative period, in addition to the routine care provided to the control group:

1. Before surgery, specialized health education was conducted for the mothers, introducing the cesarean section process, safety, preoperative preparations, surgical team information, anesthesia methods, and potential postoperative complications through videos and brochures. Psychological intervention was provided, as mothers often experienced tension and anxiety before cesarean section, which affected both their health and the nurses’ work. Nurses offered one-on-one counseling and encouragement, utilizing professional psychological knowledge and specific case explanations to patiently address the mothers’ and their families’ concerns. Through verbal encouragement, physical touch, and role modeling, mothers were guided to stabilize their mindset and build confidence in the surgery. Communication and health knowledge education were conducted with mothers and their families, with family members requested to provide spiritual comfort and encouragement to help reduce negative emotions and psychological stress. Nurses maintained a proactive attitude, continuously inquiring about the mothers’ needs and striving to meet their reasonable requests.
2. During surgery, the operating room temperature and humidity were pre-adjusted 30 min before entry to create a comfortable surgical environment. Correct positioning was assisted according to surgical needs to ensure comfort and safety, avoiding issues such as nerve or blood vessel compression due to improper positioning. Throughout the surgery, close monitoring of the mothers’ vital signs, including heart rate, blood pressure, and respiration, was conducted to ensure their safety. Any abnormalities or discomfort were immediately reported to the physician for timely intervention. Nurses also focused on protecting the mothers’ privacy and comfort, minimizing skin exposure in non-surgical areas. A series of warming measures, such as providing heating pads and blankets, were taken to prevent the mothers from feeling cold during surgery. Fluids were pre-warmed before infusion, and all operations were performed gently and precisely to minimize stress stimulation from excessive traction. At the end of the surgery, blood stains were wiped clean, and the mothers’ performance during surgery was encouraged and praised, assisting them in a smooth role transition. The mothers were then transferred to the ward after their vital signs stabilized.
3. Postoperatively, the following measures were implemented:
  - For postpartum hemorrhage care, attention was paid to observing whether mothers exhibited shock symptoms such as pallor, cold sweats, and weak pulse. Blood pressure, pulse, respiration, body temperature, uterine contractions, and vaginal bleeding volume were closely monitored. Any increase in bleeding volume or shock symptoms prompted immediate notification of the doctor and implementation of emergency measures. The healing of abdominal incisions was regularly observed, with the skin around the incisions kept dry and clean. Dressings were changed according to aseptic principles to prevent incision infections.
  - Breastfeeding guidance and care were implemented, with healthcare professionals introducing the advantages of breastfeeding to mothers and their families, encouraging breastfeeding, and instructing mothers on correct breastfeeding positions to ensure proper latching and reduce the risk of nipple fissures. Early suckling by newborns was facilitated to stimulate prolactin release and increase milk supply. Nurses guided mothers and families on recognizing infant hunger cues, effective breastfeeding techniques, and managing potential breastfeeding issues, and provided breast massage.
  - For sleep care, the ward temperature was maintained between 22 and 26°C to avoid excessive cold or heat, promoting relaxation and improving sleep quality. Nurses and family members minimized noise in the ward, avoiding the use of loud devices such as televisions and telephones, and moved gently to reduce disturbance to the mothers. Soundproofing measures, such as installing soundproof curtains or using earplugs, were taken to aid sleep. The ward lighting was kept soft to avoid direct, strong light, with nightlights used for nighttime feeding or getting up.
  - For postoperative pain care, mothers and their families were educated about uterine contraction pain and incision pain, including pain location, causes, duration, and relief methods. Nurses strengthened postoperative pain assessment and intervened using non-pharmacological analgesia (such as deep breathing relaxation, distraction, massage, music therapy) or pharmacological analgesia as prescribed, or by increasing the frequency of analgesic pump presses, based on the mothers’ pain levels, to alleviate pain.
  - Postoperative recovery interventions were implemented. After a cesarean section, family members were instructed to assist mothers with urination and ambulation to aid gastrointestinal function recovery and prevent intestinal adhesion. Family members provided protection during the mother's first ambulation to prevent falls and offer family care. Rehabilitation training was guided, starting with simple bed activities such as turning over and limb movements in the early postoperative period, gradually increasing activity levels as the body recovered, and informing mothers of rehabilitation training precautions.
  - Dietary interventions were conducted. Mothers were fasted from food and water for 6 h postoperatively and gradually resumed eating after gastrointestinal function recovery, avoiding cold, oily, and gas-producing foods. Nurses guided mothers’ diets based on their dietary preferences and clinical experience, recommending light and easily digestible foods initially, gradually transitioning to a normal diet.
  - Postoperative psychological care was provided, with attention paid to mothers’ psychological states to promptly identify and address emotional issues such as anxiety and depression. Family members were guided to provide emotional support and enhance mothers’ self-worth, avoiding excessive focus on the newborn at the expense of the mother. Nurses actively patrolled and observed, detecting subtle emotional changes in mothers and providing psychological support to help them navigate changes and challenges smoothly, avoiding bleeding due to emotional factors affecting uterine contractions.

Both groups received care until discharge.

### Data collection and outcome evaluation

The primary outcomes encompassed the degree of postoperative pain and anxiety-depression mood before and after nursing care:

1. The degree of maternal pain in both groups was assessed by the Visual Analog Scale (VAS) scores at 24, 48, and 72 h postoperatively. A 0–10 cm horizontal line was drawn on paper to represent the pain level, with a total score of 10 points. Higher scores indicated more severe pain. The evaluation was guided by researchers postoperatively.
2. Maternal anxiety and depression before and after care in both groups were assessed by the Self-Rating Anxiety Scale (SAS) and the Self-Rating Depression Scale (SDS). Both scales contained 20 items, rated based on the frequency of symptom occurrence using a Likert 1–4 point scoring method. The scores of each item were summed to obtain a raw score, which was then multiplied by 1.25, and the integer part was taken as the standard score, ranging from 25 to 100 points. For the SAS, a standard score of 50 indicated the threshold for anxiety, with scores of 50–59 indicating mild anxiety, 60–69 indicating moderate anxiety, and >69 indicating severe anxiety. The Cronbach's *α* coefficient was 0.915. For the SDS, a standard score of 53 indicated the threshold for depression, with scores of 53–62 indicating mild depression, 63–72 indicating moderate depression, and >72 indicating severe depression. The Cronbach's *α* coefficient was 0.830.

The secondary outcomes included the following indicators:

1. The mean arterial pressure (MAP) and heart rate (HR) of mothers were measured using a Mindray Bene View T5 electrocardiogram monitor 1 h before surgery and 24 h after surgery.
2. Fasting venous blood samples (5 ml) were collected from mothers before and after surgery. After centrifugation at 3000 r/min for 10 min, the serum was separated. Serum adrenaline and cortisol levels were detected using a double-antibody sandwich ABC-ELISA method. The reagent kits were purchased from Elabscience (USA), and the detection was strictly carried out according to the product instructions.
3. The time of starting lactation, 24-h postpartum lactation volume, off-bed activity time, anal exhaustion time, and hospital stay between the two groups were observed.
4. After care (at discharge), the Exercise of Self-Care Agency Scale (ESCA) was employed to assess the self-care competency of the mothers in the two groups. The scale comprised four dimensions: self-care skills (items 15–26), self-care responsibility (items 9–14), self-concept (items 1–8), and health knowledge level (items 27–43), with a total of 43 items. Each item was scored from 0 to 4 points, with a total scale score ranging from 0 to 172 points. Higher scores indicated better self-care ability. The Cronbach's *α* coefficient was 0.860.
5. The incidence of maternal complications (bleeding, infection, and delayed incision healing) between the two groups after care was compared.
6. At discharge, mothers’ satisfaction with nursing care was assessed using a self-designed satisfaction questionnaire, which included aspects such as attitude, communication, and operation. The total score ranged from 0 to 100 points, with ≥90 points indicating very satisfied, 60–90 points indicating generally satisfied, and <60 points indicating dissatisfied. The overall satisfaction rate = (number of very satisfied + generally satisfied cases)/total number of cases × 100%.

### Statistics

SPSS 20.0 statistical software was applied to analyze the data. Numeration data were depicted as [case (%)] and analyzed by the *χ*^2^ test. Measurement data were subjected to the Shapiro–Wilk test for normality. Normally distributed measurement data were expressed as ($\bar{x}$ ± *s*), with independent sample *t*-tests used for comparisons between groups and paired sample *t*-tests for comparisons within groups. Non-normally distributed measurement data were expressed as median (quartile) [M (P25, P75)], with the Mann–Whitney *U* test used for comparisons between groups and the Wilcoxon signed-rank test for comparisons within groups. The difference was statistically significant at *P* < 0.05.

## Results

### General data

The age of parturient women of the control group ranged from 23 to 32 years, with a mean of 27.27 ± 2.27 years old and an average BMI of 26.17 ± 2.59 kg/m^2^; their weeks of gestation ranged from 36 to 43 weeks; there were 26 cases of primigravid women, and 19 cases of multiparous women; the weight of newborns averaged 3.16 ± 0.35 kg. The age of parturient women of the observation group ranged from 21 to 31 years, with a mean age of 27.02 ± 2.44 years old and an average BMI of 26.36 ± 2.28 kg/m^2^; their weeks of gestation ranged from 36 to 42 weeks; there were 33 cases of primigravid women, and 12 cases of Multiparous women; the weight of newborns averaged 3.01 ± 0.42 kg. The general data of the two groups were comparable with no statistically significant difference (*P* > 0.05) (Table 1).

**Table 1.** General data between the two groups.

Indicators	The control group (*n* = 45)	The observation group (*n* = 45)	*t*-value/*Z*-value/*χ*^2^ value	*P*-value
Age (years)	27.27 ± 2.27	27.02 ± 2.44	0.492	0.624
Body mass index (kg/m2)	26.17 ± 2.59	26.36 ± 2.28	0.371	0.712
Gestation week (weeks)	39 (38,39)	39 (38,40)	−0.753	0.452
Number of births			2.411	0.121
Primigravid women	26 (57.78)	33 (73.33)		
Multiparous women	19 (42.22)	12 (26.67)		
Weight of newborns (kg)	3.16 ± 0.35	3.01 ± 0.42	1.826	0.071

### VAS scores at different times after surgery

The VAS scores of the observation group were lower than those of the control group at 24, 48, and 72 h after surgery (*P* < 0.05) (Table 2).

**Table 2.** VAS scores at different times after surgery between the two groups (point).

Grouping	24 h after surgery	48 h after surgery	72 h after surgery
The control group (*n* = 45)	3 (3,4.5)	3 (2,4)	2 (2,3)
The observation group (*n* = 45)	3 (2,4)	2 (2,2)	2 (1,2)
*Z*-value	−1.983	−3.896	−4.053
*P*-value	0.047	<0.001	<0.001

VAS, Visual Analog Scale.

### Maternal negative emotions

Prior to care, the differences in SAS and SDS scores between the two groups were not statistically significant (*P* > 0.05). After care (at discharge), both groups showed significantly lower SAS and SDS scores compared with before care, and those scores were lower in the observation group, in contrast with those in the control group (*P* < 0.05) (Table 3).

**Table 3.** Maternal negative emotions between the two groups.

Grouping	SAS scores (point)		SDS scores (point)	
	Before care	After care	Before care	After care
The control group (*n* = 45)	56.09 ± 4.18	44.80 ± 3.53^a^	58.33 ± 5.22	46.78 ± 4.57^a^
The observation group (*n* = 45)	56.13 ± 4.26	36.29 ± 2.47^a^	58.60 ± 5.31	37.38 ± 3.57^a^
*t*-value	0.050	13.240	0.240	10.880
*P*-value	0.960	<0.001	0.811	<0.001

SAS, Self-Rating Anxiety Scale; SDS, Self-Rating Depression Scale.

^a^ *P* < 0.05 vs the same group before surgery.

### Maternal cardiovascular stress indicators

Preoperatively, the differences in MAP and HR between the two groups were not statistically significant (*P* > 0.05). After surgery, when compared with those prior to surgery, the MAP and HR of the observation group were not statistically significant (*P* > 0.05), while the MAP and HR in the control group were higher than those before surgery (*P* < 0.05). After surgery, MAP and HR of the observation group were lower than those of the control group (*P* < 0.05) (Table 4).

**Table 4.** Maternal cardiovascular stress indicators between the two groups.

Grouping	MAP (mmHg)		HR (time/min)	
	Before surgery	After surgery	Before surgery	After surgery
The control group (*n* = 45)	74.56 ± 7.92	83.07 ± 9.84^a^	80.24 ± 8.79	87.60 ± 9.43^a^
The observation group (*n* = 45)	73.33 ± 4.90	75.13 ± 8.31	81.62 ± 5.98	81.47 ± 8.56
*t*-value	0.800	4.132	0.870	3.232
*P*-value	0.381	<0.001	0.387	0.002

MAP, mean arterial pressure; HR, heart rate.

^a^ *P* < 0.05 vs the same group before surgery.

### Maternal stress

Postoperatively, maternal adrenaline and cortisol levels in both the observation and control groups were higher than those before surgery, and those levels were lower in the observation group than in the control group (*P* < 0.05) (Table 5).

**Table 5.** Maternal stress between the two groups.

Grouping	Adrenaline (pg/ml)		Cortisol (ng/ml)	
	Before surgery	After surgery	Before surgery	After surgery
The control group (*n* = 45)	64.48 ± 5.78	78.22 ± 8.18^a^	92.01 ± 9.74	105.60 ± 6.10^a^
The observation group (*n* = 45)	64.29 ± 4.41	69.95 ± 2.81^a^	92.03 ± 5.92	97.27 ± 5.50^a^
*t*-value	0.175	6.415	0.012	6.827
*P*-value	0.861	<0.001	0.991	<0.001

^a^ *P* < 0.05 vs the same group before surgery.

### Postoperative recovery

The observation group had shorter lactation onset time, off-bed activity time, anal defecation time, and hospital stay, and greater 24-h postpartum lactation volume than the control group (*P* < 0.05) (Table 6).

**Table 6.** Postoperative recovery between the two groups.

Grouping	Lactation onset time (h)	24-h postpartum lactation volume (ml)	Off-bed activity time (h)	Anal defecation time (h)	Hospital stay (d)
The control group (*n* = 45)	24 (23,25)	21.38 ± 2.76	19.36 ± 5.06	21.84 ± 5.97	6 (5,8)
The observation group (*n* = 45)	22 (21,23)	30.56 ± 3.60	10.38 ± 2.22	12.87 ± 3.84	5 (4,6)
*t*-value/*Z*-value	−4.799	13.580	10.910	8.480	−4.861
*P*-value	<0.001	<0.001	<0.001	<0.001	<0.001

### Self-care competence

The scores of self-care skills, self-care responsibility, self-concept, and self-care health knowledge level of the observation group were higher than those of the control group (*P* < 0.05) (Table 7).

**Table 7.** Self-care competence between the two groups.

Grouping	Self-care skills (point)	Self-care responsibility (point)	Self-concept (point)	Self-care health knowledge level (point)
The control group (*n* = 45)	25 (22,29)	12 (10.5,15)	16 (13,18)	29 (27,32)
The observation group (*n* = 45)	33 (29.5,37.5)	15 (14.5,21)	24 (21,27)	41 (36.5,44.5)
*Z*-value	−6.185	−5.503	−6.916	−6.999
*P*-value	<0.001	<0.001	<0.001	<0.001

### Maternal complications

The total incidence of postoperative complications in the observation group was 4.44%, which was lower than that of 24.44% in the control group (*P* < 0.05) (Table 8).

**Table 8.** Maternal complications between the two groups.

Grouping	Bleeding	Infections	Delayed incision healing	Incidence
The control group (*n* = 45)	4 (8.89)	4 (8.89)	3 (6.67)	11 (24.44)
The observation group (*n* = 45)	1 (2.22)	1 (2.22)	0	2 (4.44)
*χ* ^2^ value				7.283
*P*-value				0.007

### Satisfaction with maternal care

The nursing satisfaction of the observation group was 97.78%, higher than that of the control group, which was 84.22% (*P* < 0.05) (Table 9).

**Table 9.** Satisfaction with maternal care between the two groups.

Grouping	Extremely satisfied	Generally satisfied	Dissatisfied	Total satisfaction
The control group (*n* = 45)	21 (46.67)	16 (35.56)	8 (17.78)	37 (84.22)
The observation group (*n* = 45)	36 (80.00)	8 (17.78)	1 (2.22)	44 (97.78)
*χ* ^2^ value				6.049
*P*-value				0.014

## Discussion

Cesarean delivery can result in a variety of short-term and long-term potential complications and even threaten the health of the mother and children. A new-pattern obstetrical nursing care is demonstrated to be effective in the reduction of the cesarean delivery rate [14]. This study focused on the effects of comfort care combined with family participatory care during the perioperative period of cesarean section in obstetrics and gynecology.

Pain after cesarean section is reported [15]. Effective pain management is considered a key priority in women who undergo cesarean delivery, and sub-optimal perioperative pain management is reported to have an association with chronic pain, delayed functional recovery, and raised postpartum depression [16]. In our study, we found that the VAS scores of the observation group were lower than those of the control group at 24, 48, and 72 h after cesarean section. This indicates that the model combining comfort care with family participatory care has a positive effect in alleviating postcesarean section pain. It has been reported that providing comfort care can significantly reduce pain and improve the quality of life in children undergoing tonsillectomy [17]. Additionally, family participatory care has been shown to be of great significance in improving clinical outcomes for preterm infants in neonatal intensive care units, shortening hospital stays, enhancing feeding conditions, promoting infant growth and development, and improving the psychological state of their families [18]. Meanwhile, our study found that the SAS and SDS scores of patients in the observation group were lower than those in the control group at discharge, suggesting that the combination of comfort care and family participatory care has a more pronounced regulatory effect on the psychological state of parturients, helping them better cope with the psychological stress brought about by cesarean section. Existing evidence indicates that comfort care models can be applied in maternity wards to improve the physical and mental state of parturient women and enhance nursing work efficiency and quality [19]. Mohsen *et al*.'s study stated that increasing family presence alone has no impact on patient anxiety, but family participation and interaction with the nursing team can influence anxiety levels in coronary care unit patients with heart disease and improve the nursing process [11].

As previously reported, cesarean section can induce elevated maternal stress during and after the surgery [20]. In addition, stress can result in elevated stress hormones adrenaline and cortisol, which further influence the metabolism of other hormones [21]. We found that, compared with the control group, the MAP and HR in the observation group were lower postoperatively. Furthermore, there were no significant changes in MAP and HR in the observation group compared with preoperative values, whereas both MAP and HR in the control group were higher than preoperative levels. This result suggests that the model combining comfort care with family participatory care helps reduce cardiovascular stress responses in parturient women. We also revealed that postoperatively, maternal adrenaline and cortisol levels in the observation group were lower than those in the control group, indicating that this nursing model can alleviate maternal stress responses to a certain extent. Existing data reveal that family participatory clown therapy can reduce pain, anxiety, medical fear, and crying during venous puncture in children. It can also improve compliance with venous puncture [22]; comfort nursing on the basis of the collaborative care model is reported to improve coronary heart disease patients’ health knowledge, regulate psychological status, and improve self-care capability and comfort [23]. Additionally, the results also displayed that the scores of self-care skills, self-care responsibility, self-concept, and self-care health knowledge level of the observation group were higher than those of the control group, indicating that the model combining comfort care with family participatory care can enhance the self-care ability of parturient women.

Furthermore, the observation group had lactation onset time, off-bed activity time, anal defecation time, and hospital stay, and greater 24-h postpartum lactation volume than the control group. These results suggest that the model combining comfort care with family participatory care facilitates postoperative recovery in parturients. Moreover, the overall incidence of postoperative complications in the observation group was lower than that in the control group, and nursing satisfaction was higher. This indicates that the model combining comfort care with family participatory care can prevent and reduce the occurrence of postoperative complications after cesarean section, meet the nursing needs of parturient women, and improve the quality of nursing services. Existing evidence supports that family participatory nursing is significant in the amelioration of premature infants’ clinical outcomes, which is able to shorten hospital stay, and enhance their families’ psychological status and nursing satisfaction [18]. The application of the comfort care model in maternity rooms can significantly enhance nursing work efficiency and quality [19].

It is noteworthy that the model combining comfort care with family participatory care adopted in this study and family-centered care both emphasize the crucial role of the family in nursing and actively encourage family members to participate in the care process for parturients. However, our model places greater emphasis on the concept of comfort care compared with a single family-centered care approach, focusing on alleviating maternal suffering and enhancing comfort from multiple dimensions, including physiological and psychological aspects. In contrast, family-centered care emphasizes meeting the overall needs of the family and granting families more autonomy in medical decision-making [24, 25], with certain differences in focus between the two approaches.

In summary, this research demonstrates that during the perioperative period of cesarean section, the application of comfort care combined with family participatory care in obstetrics and gynecology can effectively alleviate maternal postoperative pain, negative emotions, and stress reactions, improve maternal self-care ability and nursing satisfaction, and reduce the incidence of complications. This study lays a foundation to explore the combined effects of comfort care and family participatory care during the perioperative period of cesarean section in obstetrics and gynecology. However, this study is based on limited clinical data collected from a single hospital and lacks long-term follow-up studies on the effects on parturients and infants. Future research could conduct multicenter, large-sample studies to comprehensively evaluate the long-term effects and benefits of this nursing model and further assess its clinical efficacy.

## Data Availability

The experimental data used to support the findings of this study are available from the corresponding author upon request.
